# The effect of progesterone use in the first trimester on fetal nuchal translucency

**DOI:** 10.4274/jtgga.2017.0056

**Published:** 2018-03-01

**Authors:** Müberra Namlı Kalem, Ziya Kalem, Batuhan Bakırarar, Ali Ergün, Timur Gürgan

**Affiliations:** 1 Clinic of Obstetrics and Gynecology, Liv Hospital, Ankara, Turkey; 2 Gürgan Clinic IVF and Women Health Center, Ankara, Turkey; 3 Department of Biostatistic, Ankara University School of Medicine, Ankara, Turkey

**Keywords:** Progesterone, nuchal translucency, maternal weight, prenatal screening test, assisted reproductive technology pregnancies

## Abstract

**Objective:**

To evaluate the possible association between progesterone use in the first trimester of pregnancy and fetal nuchal translucency (NT).

**Material and Methods:**

This is an observational case-control study, which was conducted with patients who underwent nuchal scans between March 2015 and February 2016 and consequently delivered live and healthy babies. The study group was composed of assisted reproductive technology pregnancies and used intravaginal progesterone 180 mg/day until gestational week 12. The control group comprised pregnant women who became pregnant spontaneously without using any progesterone preparation in the first trimester.

**Results:**

One hundred sixty-four (57.5%) of 285 patients were in the control group and 121 (42.5%) were in the progesterone group. Age, bodyweight, gravidity, and parity number of previous births and abortus, gestational week, crown-rump lengths, free β-human chorionic gonadotropin, pregnancy-associated plasma protein A, and NT values of the progesterone and control groups were recorded and we investigated whether there was a statistically significant difference between the two groups in terms of these parameters; maternal weight was found to be higher in the progesterone group than in the control group and the difference between the groups was statistically significant (p=0.019 and p=0.025). Whether the difference in NT was caused by the effect of maternal weight was investigated using the covariance analysis test and maternal weight was not found to be statistically significant in the model (p=0.284).

**Conclusion:**

Fetal NT was increased in the progesterone group compared with the untreated group in healthy pregnancies.

## Introduction

The first trimester combined test, which was first introduced in the 1990s, is a current test for the evaluation of fetal chromosomal anomaly (1, 2, 3). Data used to calculate risk in this test are fetal nuchal translucency (NT), maternal serum free b-human chorionic gonadotropin (b-hCG), and pregnancy-associated plasma protein-A (PAPP-A) between weeks 11 and 14 of pregnancy, in addition to maternal age (4,5). Giorlandino et al. (6), in their study published in 2015, hypothesized that progesterone could lead to abnormal blood flow patterns, and thus to increased NT. The authors, however, concluded that the results of the screening test were not affected.

NT, a sensitive marker in screening Down syndrome, is still considered controversial due to high false-positive rates (7, 8, 9). In addition, progesterone has been used widely for prophylaxis and treatment of abortus in cases of threatened miscarriage in the first trimester and in pregnancies conceived after assisted reproductive technology (ART) treatment (10, 11, 12, 13). In this case, if the thickness of NT changes in patients using progesterone, the question is raised as to whether this condition increases the false positivity rate when screening for Down syndrome in the first trimester.

The aim of this study was to evaluate the possible association between progesterone use in the first trimester of pregnancy and fetal NT in healthy pregnancies without any known risk factors.

## Material and Methods

This is an observational case-control study, which was conducted with patients who underwent nuchal scans between March 2015 and February 2016 and consequently delivered live and healthy babies. All patients who participated in the study were between 11.0 and 13.6 weeks’ gestation, which are the suitable gestational weeks for the first-trimester Down syndrome screening test, and their fetal crown-rump lengths (CRL) were between 41 mm and 84 mm. The study group was composed of patients who became pregnant with ART in a private *in vitro* fertilization (IVF) clinic and whose pregnancies were monitored; these patients used intravaginal progesterone 180 mg/day (Crinone gel; Serono, İstanbul, Turkey) until gestational week 12, as used in the monitoring of all ART pregnancies. The control group comprised pregnant women who became pregnant spontaneously without using any progesterone preparation in the first trimester. All the patients used folic acid, iron or multivitamin preparations, and those who were using other medications were not included in the study. Patients who were found to have high risk (cut-off 1/300) in the nuchal scan, whose NT values were above 2.5 mm and in whom any congenital or chromosomal anomalies were detected in amniocentesis or in the monitoring, and women who had systemic disorders such as diabetes and hypertension were excluded. Patients who had plural pregnancy or bleeding in the first trimester were also not included in the study. 

In all patients, age, body weight, gravidity and parity, number of previous births and abortus, gestational week, CRL, PAPP-A, free b-hCG and NT values, presence/lack of nasal bone, and whether the patient was a smoker were recorded. For the patients’ obstetric ultrasonographic (USG) evaluations, and CRL and NT measurements, a General Electric Voluson 730 Expert (GE Medical Systems, Kretztechnik, Zipf, Austria) with a 3D/4D transabdominal multifrequency probe was used. Two expert physicians performed the USG evaluations.

### Statistical analysis

All statistical analyses were performed using the SPSS for Windows 11.5 software program (SPSS Inc., Chicago, IL). Compatibility of data with normal distribution was examined graphically and with the Kolmogorov-Smirnov test. For the quantitative variables, mean ± standard deviation and median (minimum-maximum) were used, and for the categorical variables, numbers (percentage) were used as descriptors in the study. When determining whether there was a statistically significant difference between the categories using qualitative variables with two categories in terms of quantitative variables, Student’s t-test was used if the assumption of normal distribution was met; if not, the Mann-Whitney U test was used. The chi-square test was used to examine the relationship between the two categorical variables. Spearman’s rank correlation coefficient was used to see if there was a statistically significant relationship between two quantitative variables because at least one of the variables did not meet the assumption of normal distribution. Covariance analysis (ANCOVA) was used to establish whether one or more continuous independent parameters had any impact on the dependent parameter. ROC analysis was used to find the discriminative factors between the groups. The significance level was set at p=0.05.

## Results

One hundred sixty-four (57.5%) of the 285 patients were in the non-progesterone group and 121 (42.5%) were in the progesterone group. The age, body weight, gravidity and parity, number of previous births and abortus, gestational week, CRL, free b-hCG, PAPP-A and NT values of the progesterone and non-progesterone groups are shown in Table 1. Whether there was a statistically significant difference between the two groups regarding these parameters was examined. NT values were found to be higher in the progesterone group than in the non-progesterone group, and the difference between the groups were found to be statistically significant (p=0.019). Whether the difference in NT was caused by the effect of maternal weight was investigated using the ANCOVA test and it was not found to be statistically significant (p=0.284); it can be concluded that maternal weight does not affect NT. CRL values were found to be higher in the non-progesterone group than in the progesterone group, and the difference between the groups was found to be statistically significant (p=0.026). The parameter of gestational week was found to be higher in the non-progesterone group than in the progesterone group, and the difference between the groups was statistically significant (p=0.006).

The number of previous abortus was found higher in the progesterone group than in the non-progesterone group, and the difference between the groups was statistically significant (p=0.019). Maternal weight was found to be higher in the progesterone group than in the non-progesterone group, and the difference between the groups was statistically significant (p=0.025) (Table 1).

No statistically significant difference was found between the two groups regarding smoking status (p=0.558). The rate of non-smokers was 95.7% in the non-progesterone group and 94.2% in the progesterone group. 

No statistically significant difference was found between the two groups with regards the presence/lack of nasal bone (p=0.463). The rate of presence of nasal bone was 93.9% in the non-progesterone group and 95.9% in the progesterone group. 

We investigated whether fetal NT measurement was related to maternal and fetal parameters and no statistically significant relationship was found between NT and these parameters (Table 2), and no statistically significant relationship was found between NT, smoking status of the mother, and the presence/lack of nasal bone (p=0.579 and p=0.950, respectively).

## Discussion

The effect of first-trimester progesterone use on NT measurement was investigated in our study and it was seen that NT values were statistically and significantly higher in the progesterone group than in the non-progesterone group. 

Giorlandino et al. (6) (2015) revealed that exogenous progesterone intake in the first trimester had an enhancing effect on fetal NT. In the same study, it was stated that the increase in NT did not change the results of the first-trimester fetal aneuploidy screening test and was independent of progesterone use. Nevertheless, a subsequent correspondence via the editor of the journal noted that the increase in NT was only in week 11 and did not include weeks 12 and 13 (14). On the other hand, it was stated that different preparations and uses might render the evaluation unhealthy. In another criticism, it was argued that bleeding in the progesterone group with the risk of miscarriage might change fetal circulation and have an impact on NT. Based on these criticisms, we planned to conduct our study with patients who used the same preparation for the same duration and had not had bleeding in pregnancy. Progesterone gel is administered intravaginally to all women with post-ART pregnancies until week 12 in our collaborating IVF clinic; our study group was selected from among those women. No data regarding whether NT values were affected in ART pregnancies were observed in our literature review (15). Although it was reported in the literature that PAPP-A values were lower in ART pregnancies, PAPP-A values did not differ between the groups in our study (16). No relationship between NT values and gestational week was found; due to that fact and the limited number of patients, the progesterone-NT relationship was not evaluated by gestational weeks.

Keçecioğlu et al. (17) (2016) studied the subject with a group with low risk of miscarriage and reported that progesterone usage increased NT values and this change was positively related to the duration of progesterone usage. In our study, no such comparison was made as the progesterone usage durations and dosage remained fixed.

The relationship between NT values and maternal and fetal parameters was investigated in our study but no correlation was found. As maternal age advances, chromosomal anomaly incidence and consequently NT values increase; however, no study was observed in the literature to reveal the relationship between maternal age and NT value in fetuses with no chromosomal anomalies (18, 19, 20). It is normal that no relationship was established because no pregnancies with anomalies were included in our study. Ferreira et al. (21) (2015) investigated whether maternal age, which is known to affect NT measurement values by physicians who perform USG NT evaluations, and it was reported that measurements by expert ultrasonographers had no impact on the values, contrary to those of inexperienced operators. In our study, the physicians who evaluated NT with USG knew the maternal ages.

The most important limitation of this study is that some parameters differed statistically and significantly in the progesterone and non-progesterone groups. As for the differing parameters, the number of abortus being higher in the progesterone group is an expected result because that group included only ART pregnancies and this would not affect the NT value. Again, the fewer gestational weeks and lower CRL values in this group would not cause an increase in NT values. It was investigated whether there was a relationship between higher maternal weight and higher NT values in the progesterone group, and it was concluded that the maternal weight parameter did not affect the NT parameter. As we currently do not know how ART affects NT values in the first trimester, the study design would be more appropriate if all patients were similar in terms of the ways they became pregnant; this is also a limitation of our study.

In conclusion, it was seen in our study that fetal NT was increased in the first-trimester progesterone group compared with the untreated group. These data need to be confirmed by future studies with larger groups of patients such that it can be reflected in prenatal screening tests.

## Figures and Tables

**Table 1:**
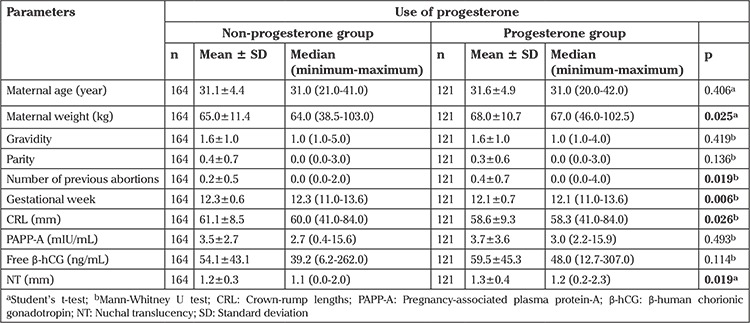
Comparison of age, body weight, gravidity and parity, number of previous births and abortions, gestational week, crown-rump lengths, pregnancy-associated plasma protein-A, free β-human chorionic gonadotropin and nuchal translucency values in the progesterone and non-progesterone groups

**Table 2:**
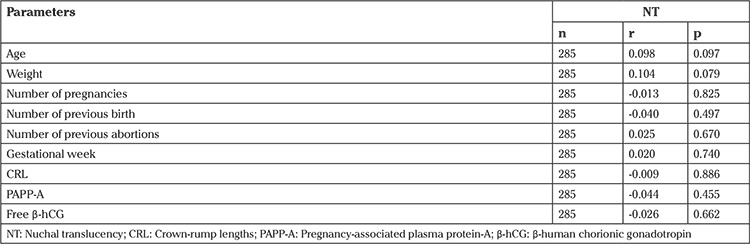
Relationship between fetal nuchal translucency measurement and maternal and fetal parameters
